# The effects of reading literary fiction on the measurement and development of mentalization skills among schizophrenic patients

**DOI:** 10.1192/j.eurpsy.2022.413

**Published:** 2022-09-01

**Authors:** J. Fekete, T. Tényi, Z. Pótó, E. Varga, R. Herold

**Affiliations:** 1 University of Pécs, Department Of Languages For Biomedical Purposes And Communication, Pécs, Hungary; 2 Medical Faculty, University of Pécs, Hungary, Department Of Psychiatry And Psychotherapy, Pécs, Hungary; 3 University of Pécs, Faculty Of Humanities And Social Sciences, Pécs, Hungary

**Keywords:** theory of mind, schizophrenia, mentalisation, reading, literary fiction

## Abstract

**Introduction:**

Following the mentalization of interpersonal relations can be improved through reading for which the influence of literary fiction can also serve as a model. Schizophrenia is characterized by extensive deficits in mentalization, and the amelioration of these impairments is a major focus in psychosocial treatment research. Reading literature can be a potential tool in improving mentalizing skills.

**Objectives:**

We aimed to examine and compare healthy participants with patients living with schizophrenia, focusing on measuring mentalizing skills and the impact of reading literary fiction on their mentalization skills.

**Methods:**

47 persons with schizophrenia in remission and 48 healthy controls were assessed and compared with Short Story Task (SST) a new measurement of ToM. SST proved to be a sensitive tool, to individual differences. After reading the short story “The End of Something” (Hemingway) a structured interview was done with 14 questions.

**Results:**

We found that patients with schizophrenia performed significantly worse in their ToM scores compared to healthy controls (ANOVA test, p<0,05 ). Previous reading experiences correlated significantly with mentalizing scores not just in healthy controls (Independent Samples T-test, p<0,05) but also in patients with schizophrenia. ToM scores were twice as high among those who had prior reading experiences in the schizophrenia group ((MS= 3,91, SD=3,166, M=8,08, SD=4,542; p<0,05, t=-3,509).

**Conclusions:**

We found that mentalization skills could be improved by regular reading. Our results could also be influenced by several other factors such as empathy skills, identification with the characters etc. Our results and conclusions are in line with the results of international research on this topic.

**Disclosure:**

No significant relationships.

